# Supplementary material for: Population structure and antibiotic resistance of swine extraintestinal pathogenic Escherichia coli from China
Source: Nat Commun. 2024 Jul 10;15:5811. doi: 10.1038/s41467-024-50268-2 (PMC11237156; doi:10.1038/s41467-024-50268-2)
Supplement: Supplementary file 1 — Supplementary Information [file 41467_2024_50268_MOESM1_ESM.pdf]

Supplementary Information for:

Population structure and antibiotic resistance of swine extraintestinal pathogenic *Escherichia coli* from China

Supplementary Figures:

Supplementary Figure 1 | The isolation time and tissue source of swine-derived ExPEC isolates in China.

Supplementary Figure 2 | The pie charts illustrating the distribution of 499 ExPEC isolates across different MLSTs (a) and serotypes (b).

Supplementary Figure 3 | Distribution of genes encoding virulence factors among swine-derived ExPEC isolates.

Supplementary Figure 4 | The prevalence of genetic determinants of antibiotic resistance in four common STs of ExPEC.

Supplementary Figure 5 | Distribution of genetic determinants responsible for fluoroquinolone resistance among swine-derived ExPEC isolates.

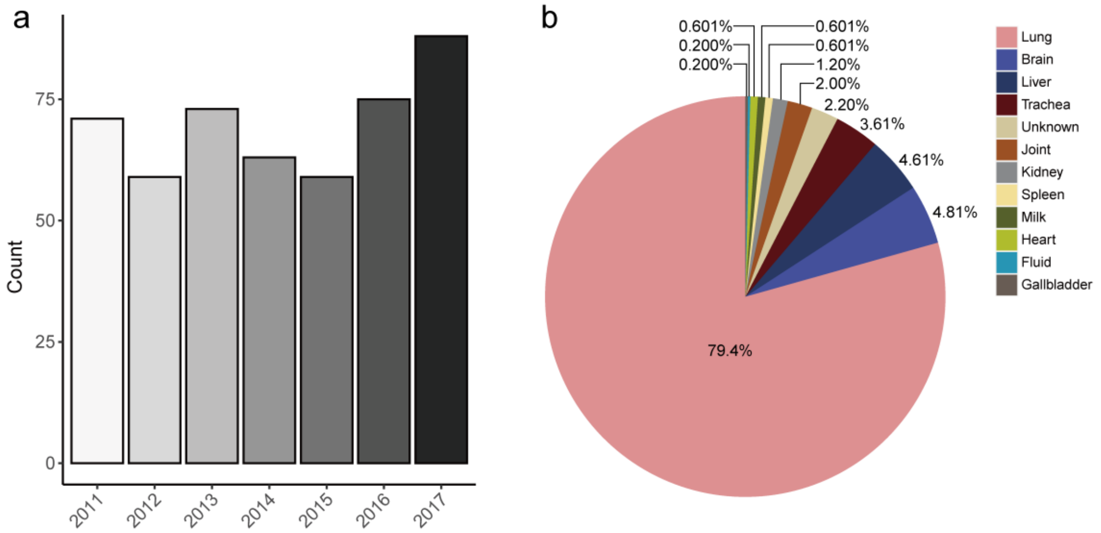

Supplementary Figure 1 | The isolation time and tissue source of swine-derived ExPEC isolates in China. a Bar plot illustrating the annual count of isolated strains. **b** Pie chart depicting the distribution of isolates from various tissues. Source data are provided as a Source Data file.

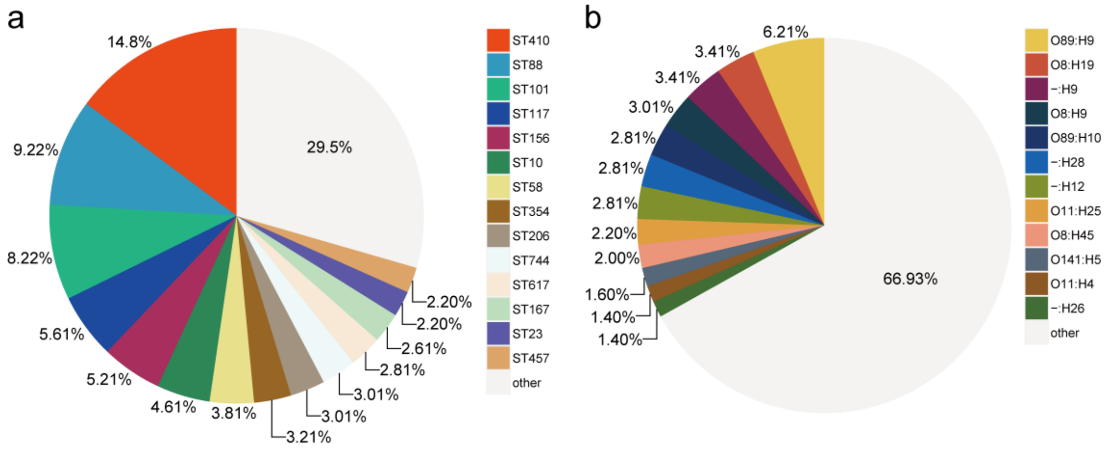

Supplementary Figure 2 | The pie charts illustrating the distribution of 499 ExPEC isolates across different MLSTs (a) and serotypes (b). Source data are provided as a Source Data file.

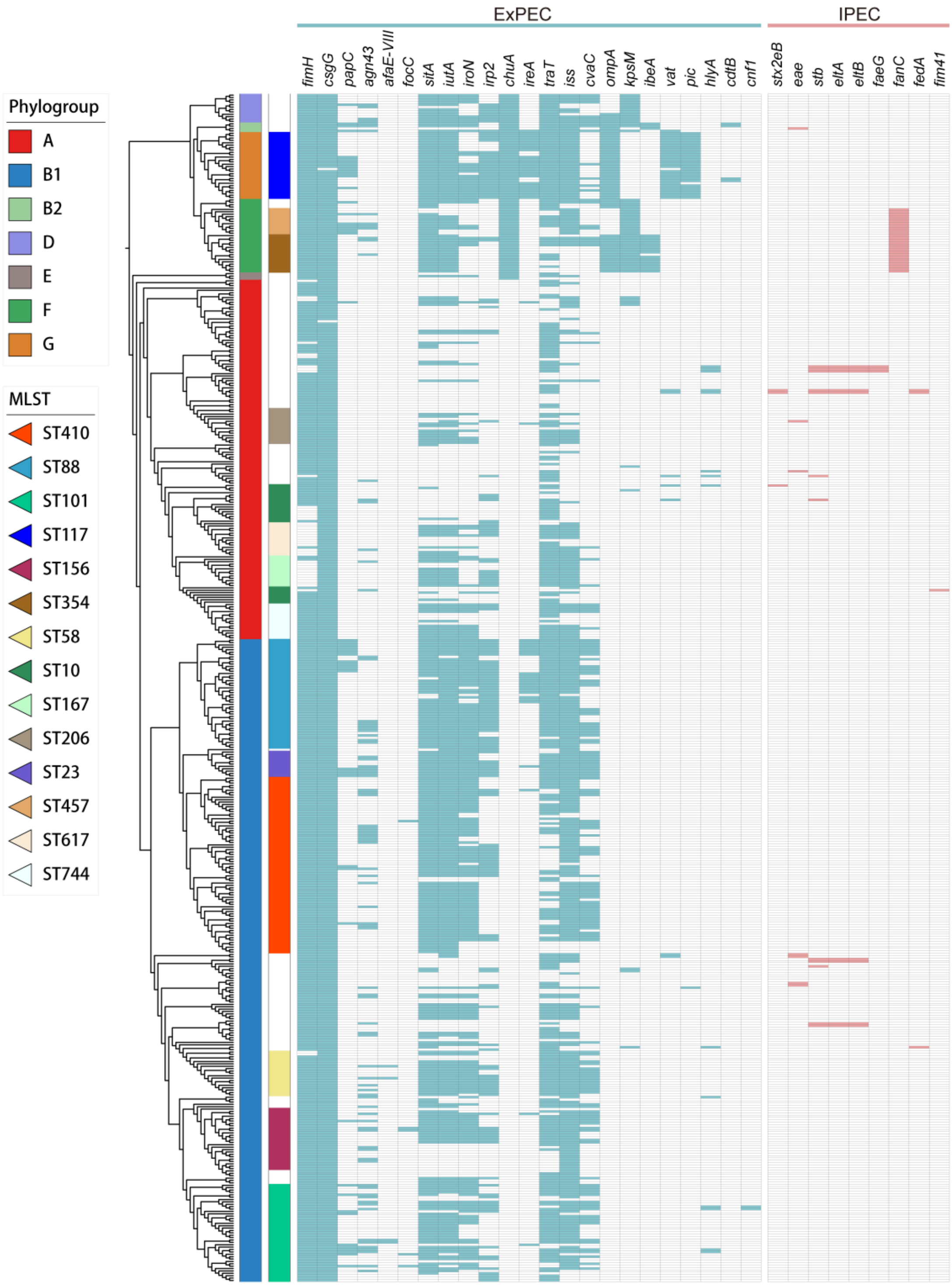

Supplementary Figure 3 | Distribution of genes encoding virulence factors among swine-derived ExPEC isolates. The tree on the left is a maximum likelihood phylogenetic tree of 499 strains with ignored branch lengths. From left to right, the first column displays the phylogroups, and the second column showcases prevalent STs. The remaining columns depict the presence and absence of each gene in these isolates. For operon, only visualize one target gene. Source data are provided as a Source Data file.

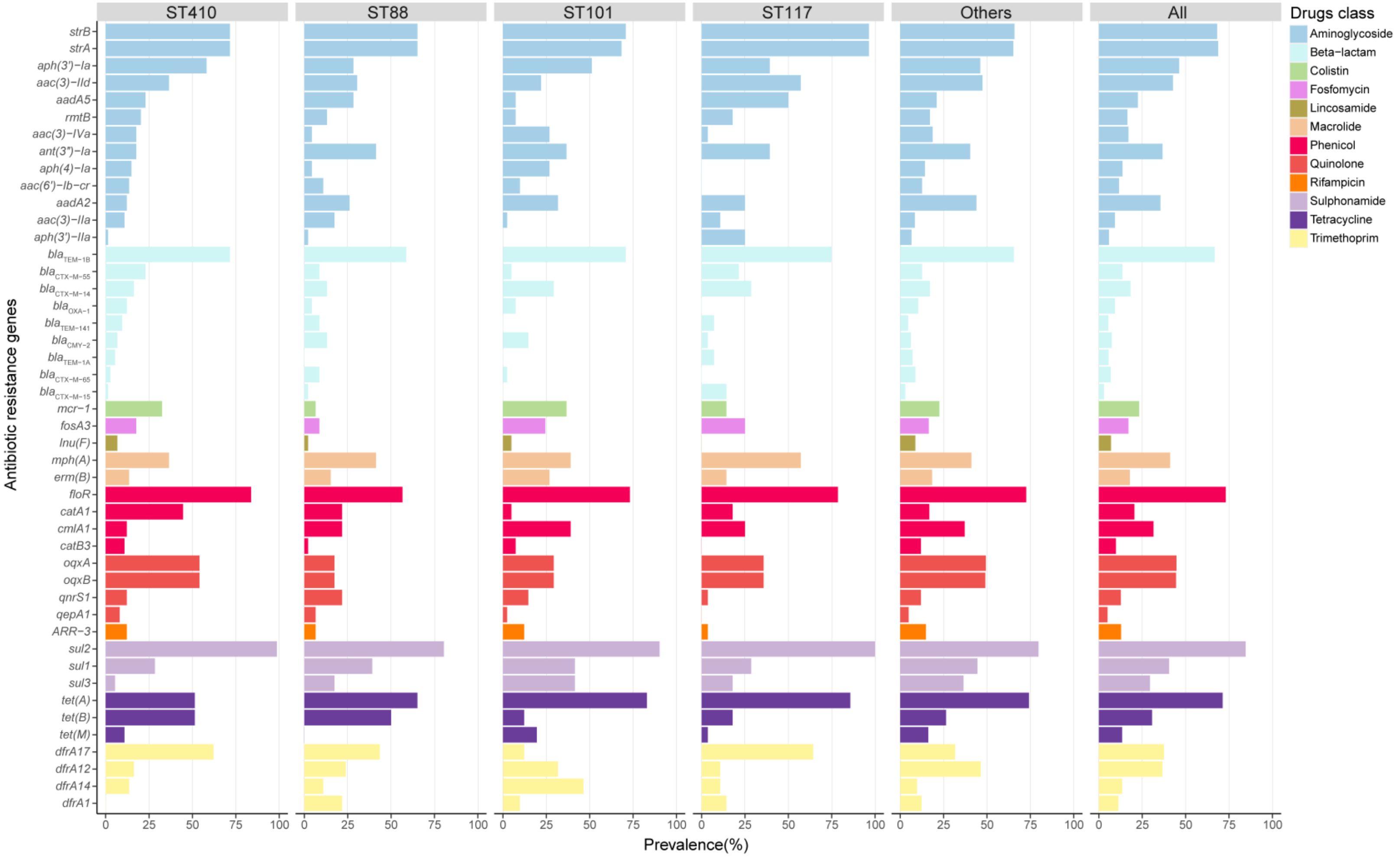

Supplementary Figure 4 | The prevalence of genetic determinants of antibiotic resistance in four common STs of ExPEC. Source data are provided as a Source Data file.

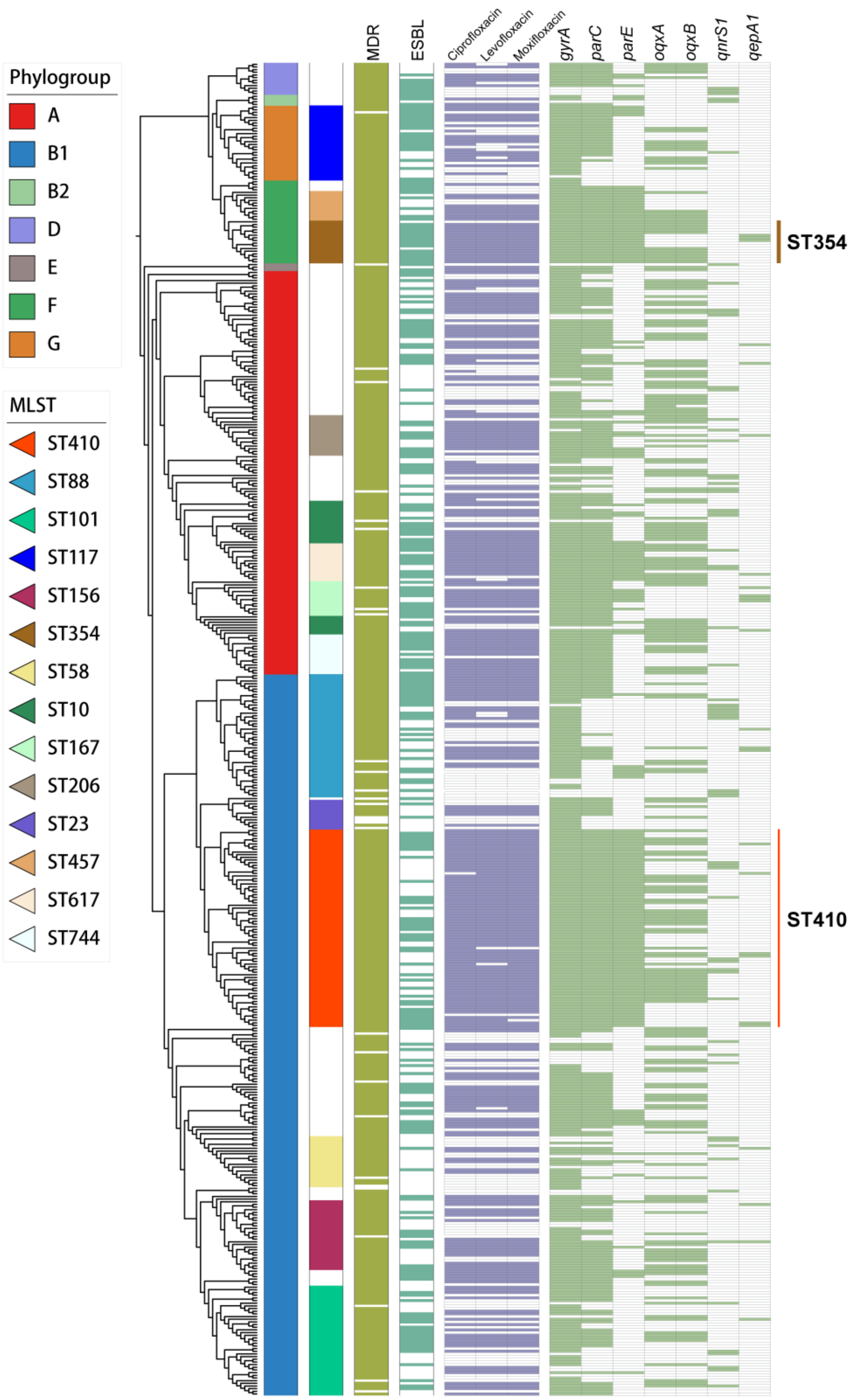

Supplementary Figure 5 | Distribution of genetic determinants responsible for fluoroquinolone resistance among swine-derived ExPEC isolates. The tree on the left represents a maximum likelihood phylogenetic tree of 499 strains, with branch lengths ignored. The first column illustrates the phylogroups from left to right, while the second column highlights the predominant STs. The subsequent columns represent the presence or absence of each genetic determinants across these isolates. Source data are provided as a Source Data file.
