# Supplementary material for: Population structure and antibiotic resistance of swine extraintestinal pathogenic Escherichia coli from China
Source: Nat Commun. 2024 Jul 10;15:5811. doi: 10.1038/s41467-024-50268-2 (PMC11237156; doi:10.1038/s41467-024-50268-2)
Supplement: Supplementary file 3 — Description of Additional Supplementary Files [file 41467_2024_50268_MOESM3_ESM.pdf]

Description of Additional Supplementary Files

File Name: Supplementary Data 1
Description: Details of 499 swine-derived ExPEC isolates in this study.

File Name: Supplementary Data 2
Description: The result of cgMLST analysis for swine ExPEC with a threshold of ≤10 allele differences.

File Name: Supplementary Data 3
Description: The matrix representing the presence or absence of virulence factors (VFs) in 499 ExPEC genomes.

File Name: Supplementary Data 4
Description: The matrix representing the presence or absence of resistance to 20 antibiotics inferred from susceptibility tests.

File Name: Supplementary Data 5
Description: The matrix representing the presence or absence of ARGs and point mutations contributing to antibiotic resistance in 499 ExPEC genomes.

File Name: Supplementary Data 6
Description: The matrix representing the presence or absence of ESBL genes in 499 ExPEC genomes.

File Name: Supplementary Data 7
Description: Compositional profiles of ARGs.

File Name: Supplementary Data 8
Description: Co-occurrence of pair of ARGs.

File Name: Supplementary Data 9
Description: Annotation information for 20 complete genomes.

File Name: Supplementary Data 10
Description: The count of ARGs identified on chromosomes and plasmids, respectively.

File Name: Supplementary Data 11
Description: Integrons and associated ARGs in 20 complete genomes.

File Name: Supplementary Data 12
Description: Primer sequences used for amplifying virulence genes.

File Name: Supplementary Data 13
Description: The MIC values and breaking points of 20 tested antibiotics.

File Name: Supplementary Data 14
Description: Summary statistics of filtered long-reads.
