# Supplementary material for: Population structure and antibiotic resistance of swine extraintestinal pathogenic Escherichia coli from China
Source: Nat Commun. 2024 Jul 10;15:5811. doi: 10.1038/s41467-024-50268-2 (PMC11237156; doi:10.1038/s41467-024-50268-2)
Supplement: Supplementary file 5 — Reporting Summary [file 41467_2024_50268_MOESM5_ESM.pdf]

nature portfolio

Corresponding author(s): Jinshui Zheng and Chen Tan

Last updated by author(s): Jun 20, 2024

Reporting Summary

Nature Portfolio wishes to improve the reproducibility of the work that we publish. This form provides structure for consistency and transparency in reporting. For further information on Nature Portfolio policies, see our Editorial Policies and the Editorial Policy Checklist.

Statistics

For all statistical analyses, confirm that the following items are present in the figure legend, table legend, main text, or Methods section.

n/a	Confirmed	
☐	☒	The exact sample size (*n*) for each experimental group/condition, given as a discrete number and unit of measurement
☐	☒	A statement on whether measurements were taken from distinct samples or whether the same sample was measured repeatedly
☒	☐	The statistical test(s) used AND whether they are one- or two-sided<br>*Only common tests should be described solely by name; describe more complex techniques in the Methods section.*
☒	☐	A description of all covariates tested
☒	☐	A description of any assumptions or corrections, such as tests of normality and adjustment for multiple comparisons
☒	☐	A full description of the statistical parameters including central tendency (e.g. means) or other basic estimates (e.g. regression coefficient) AND variation (e.g. standard deviation) or associated estimates of uncertainty (e.g. confidence intervals)
☒	☐	For null hypothesis testing, the test statistic (e.g. *F*, *t*, *r*) with confidence intervals, effect sizes, degrees of freedom and *P* value noted<br>*Give P values as exact values whenever suitable.*
☒	☐	For Bayesian analysis, information on the choice of priors and Markov chain Monte Carlo settings
☒	☐	For hierarchical and complex designs, identification of the appropriate level for tests and full reporting of outcomes
☒	☐	Estimates of effect sizes (e.g. Cohen's *d*, Pearson's *r*), indicating how they were calculated

Our web collection on statistics for biologists contains articles on many of the points above.

Software and code

Policy information about availability of computer code

Data collection	The short-reads from Illumina sequencing were de novo assembled using SPAdes v3.14.1. The long-reads from Nanopore sequencing were de novo assembled using Flye v2.8.3-b1695.
Data analysis	Prokka v1.14.6 was used to annotate genomes. SRST2 v0.2.0 was used to predict sequence type of each strain. ECTyper was used to predict serotype of each strain. Virulence factors (VFs) and antibiotic resistance genes (ARGs) were identified using ABRicate v1.0.1 with Ecoli_VF database and ResFinder database, respectively. Chromosomal point mutations contributing to antibiotic resistance were detected using ResFinder v4.1.5 based on the PointFinder database. Roary v3.13.0 was used to conduct pangenome analysis. SNP-sites v2.5.1 was used to extract the single nucleotide polymorphisms (SNPs). RAxML v8.2.12 was used to construct the phylogenetic tree. cgMLSTFinder v.1.2.0 was used to perform core genome multi-locus sequence typing (cgMLST) analysis with the Enterobase Escherichia/Shigella cgMLST v1 scheme. MOB-suite v3.0.3 was used to predict the replicon family and transferability of plasmids. Insertion sequences (ISs) were identified using ABRicate v1.0.1 with ISfinder database. Integrons were detected using IntegronFinder v2.0.3. Sourmash v3.3.0 was used to calculate Jaccard indices between pairs of plasmids. Detection of Genomic Islands on the chromosomes was accomplished using a custom Python script. BWA v0.7.17-r1188 was used to align short reads from each strain to the plasmid genomes. Bedtools v2.30.0 was used to compute coverage for sequence alignments. ggplot2 v3.3.6 and UpSetR v1.4.0 were used to visualize phenotype and genotype data in R. iTOL v5 was used to visualize phylogenetic trees. Cytoscape v3.8.0 was used to generate the co-occurrence network of ARGs. The ARGs and ISs arrow maps were created using gggenes v0.4.1. Heatmaps to visualize Jaccard similarity matrix of plasmid genomes were generated using pheatmap v1.0.12. Adobe Illustrator was used for figures retouching.<br>All custom Python and R codes have been uploaded to GitHub: https://github.com/xdli009/Swine_ExPEC_genomes_analysis.git.

For manuscripts utilizing custom algorithms or software that are central to the research but not yet described in published literature, software must be made available to editors and reviewers. We strongly encourage code deposition in a community repository (e.g. GitHub). See the Nature Portfolio guidelines for submitting code & software for further information.

Data

Policy information about availability of data

All manuscripts must include a data availability statement. This statement should provide the following information, where applicable:

- Accession codes, unique identifiers, or web links for publicly available datasets
- A description of any restrictions on data availability
- For clinical datasets or third party data, please ensure that the statement adheres to our policy

All clean short-read and long-read sequence data, draft genomes, and complete genomes generated in this study have been deposited in the NCBI under BioProject ID PRJNA1044843. BioSample accession number for each strain are provided in Supplementary Data 1.

Research involving human participants, their data, or biological material

Policy information about studies with human participants or human data. See also policy information about sex, gender (identity/presentation), and sexual orientation and race, ethnicity and racism.

Reporting on sex and gender	Not human subject research.
Reporting on race, ethnicity, or other socially relevant groupings	Not human subject research.
Population characteristics	Not human subject research.
Recruitment	Not human subject research.
Ethics oversight	Not human subject research.

Note that full information on the approval of the study protocol must also be provided in the manuscript.

Field-specific reporting

Please select the one below that is the best fit for your research. If you are not sure, read the appropriate sections before making your selection.

☒ Life sciences ☐ Behavioural & social sciences ☐ Ecological, evolutionary & environmental sciences

For a reference copy of the document with all sections, see nature.com/documents/nr-reporting-summary-flat.pdf

Life sciences study design

All studies must disclose on these points even when the disclosure is negative.

Sample size	To understand the the genomic diversity and antibiotic resistance of swine extraintestinal pathogenic Escherichia coli, we randomly selected 50-90 isolates for each year from 2011 to 2017 from diseased tissues of pigs from different piggeries in China. This sample size range was chosen to ensure an adequate representation of the population while considering feasibility and cost constraints. In total, 499 E. coli strains were collected for this study.
Data exclusions	No data were excluded from the analyses.
Replication	Antibiotic susceptibility testing methods and breaking points followed the recommended microbroth dilution protocol (CLSI M100, 28th Edition) of the Clinical & Laboratory Standards Institute (CLSI, United States). Each antibiotic test was conducted with three replicates, yielding consistent results across replicates.
Randomization	This is not relevant to our study as all strains were considered as a single group.
Blinding	Biological samples were randomly collected without prior knowledge of the antibiotic resistance phenotypes.

Reporting for specific materials, systems and methods

We require information from authors about some types of materials, experimental systems and methods used in many studies. Here, indicate whether each material, system or method listed is relevant to your study. If you are not sure if a list item applies to your research, read the appropriate section before selecting a response.

Materials & experimental systems

n/a	Involved in the study
☒	☐ Antibodies
☒	☐ Eukaryotic cell lines
☒	☐ Palaeontology and archaeology
☒	☐ Animals and other organisms
☒	☐ Clinical data
☒	☐ Dual use research of concern
☒	☐ Plants

Methods

n/a	Involved in the study
☒	☐ ChIP-seq
☒	☐ Flow cytometry
☒	☐ MRI-based neuroimaging

Plants

Seed stocks

Report on the source of all seed stocks or other plant material used. If applicable, state the seed stock centre and catalogue number. If plant specimens were collected from the field, describe the collection location, date and sampling procedures.

Novel plant genotypes

Describe the methods by which all novel plant genotypes were produced. This includes those generated by transgenic approaches, gene editing, chemical/radiation-based mutagenesis and hybridization. For transgenic lines, describe the transformation method, the number of independent lines analyzed and the generation upon which experiments were performed. For gene-edited lines, describe the editor used, the endogenous sequence targeted for editing, the targeting guide RNA sequence (if applicable) and how the editor was applied.

Authentication

Describe any authentication procedures for each seed stock used or novel genotype generated. Describe any experiments used to assess the effect of a mutation and, where applicable, how potential secondary effects (e.g. second site T-DNA insertions, mosiacism, off-target gene editing) were examined.
